# A mobile-assisted voice condition analysis system for Parkinson’s disease: assessment of usability conditions

**DOI:** 10.1186/s12938-021-00951-y

**Published:** 2021-11-21

**Authors:** Javier Carrón, Yolanda Campos-Roca, Mario Madruga, Carlos J. Pérez

**Affiliations:** 1 Departamento de Matemáticas, Universidad de Extremadura, Cáceres, Spain; 2 Departamento de Tecnología de los Computadores y las Comunicaciones, Universidad de Extremadura, Cáceres, Spain

**Keywords:** Acoustic features, Machine learning, mPower database, Parkinson’s disease, Speech processing, Voice condition analysis system

## Abstract

**Background and objective:**

Automatic voice condition analysis systems to detect Parkinson’s disease (PD) are generally based on speech data recorded under acoustically controlled conditions and professional supervision. The performance of these approaches in a free-living scenario is unknown. The aim of this research is to investigate the impact of uncontrolled conditions (realistic acoustic environment and lack of supervision) on the performance of automatic PD detection systems based on speech.

**Methods:**

A mobile-assisted voice condition analysis system is proposed to aid in the detection of PD using speech. The system is based on a server–client architecture. In the server, feature extraction and machine learning algorithms are designed and implemented to discriminate subjects with PD from healthy ones. The Android app allows patients to submit phonations and physicians to check the complete record of every patient. Six different machine learning classifiers are applied to compare their performance on two different speech databases. One of them is an in-house database (UEX database), collected under professional supervision by using the same Android-based smartphone in the same room, whereas the other one is an age, sex and health-status balanced subset of mPower study for PD, which provides real-world data. By applying identical methodology, single-database experiments have been performed on each database, and also cross-database tests. Cross-validation has been applied to assess generalization performance and hypothesis tests have been used to report statistically significant differences.

**Results:**

In the single-database experiments, a best accuracy rate of 0.92 (AUC = 0.98) has been obtained on UEX database, while a considerably lower best accuracy rate of 0.71 (AUC = 0.76) has been achieved using the mPower-based database. The cross-database tests provided very degraded accuracy metrics.

**Conclusion:**

The results clearly show the potential of the proposed system as an aid for general practitioners to conduct triage or an additional tool for neurologists to perform diagnosis. However, due to the performance degradation observed using data from mPower study, semi-controlled conditions are encouraged, i.e., voices recorded at home by the patients themselves following a strict recording protocol and control of the information about patients by the medical doctor at charge.

## Introduction

Parkinson’s disease (PD) is an up-to-now incurable neurodegenerative disorder that mainly, but not exclusively, affects the motor system. It is the most relevant neurodegenerative disorder after Alzheimer’s disease, but with a faster growth. The Global Burden of Disease study projects to reach 13 million people affected by PD in 2040 [10].

PD is typically diagnosed by a neurologist when certain motor symptoms become clinically evident, in particular when bradykinesia occurs along with rigidity or tremor. Early diagnosis is key to improve quality of life of people suffering from PD. However, in the European survey presented by Bloem and Stocchi [6], diagnosis time after the first symptoms’ onset was above 2 years in 11.8% of the patients. Misdiagnoses are also common and can be as high as 25% when the practitioners have limited clinical experience in PD [26]. The situation is critical in developing countries, where many patients remain undiagnosed [11]. Therefore, new tools seem necessary to obtain an early diagnosis.

Subjects with PD suffer from speech impairment [8]. This leads to consider automatic analysis of voice recordings as a potential tool to aid diagnosis. Different vocal tasks, focused on phonation, articulation, prosody, and cognitive–linguistic aspects have been used for the detection of PD through voice. The most used vocal task is the sustained phonation of the /a/ vowel due to its simplicity and ubiquity in different languages [30, 46]. Previous works have used a wide variety of acoustic features extracted from this type of speech recordings. For example, perturbation measures (such as Jitter or Shimmer [50]), noise measures (for instance, the harmonic-to-noise ratio (HNR) [20]), spectral and cepstral features [37], and several features based on nonlinear analysis [50], among others.

Also, diadochokinesis test recordings studying articulatory tasks [28, 41], prosodic features extracted from reading texts and spontaneous speech [19, 53], and even combinations of different vocal tasks [43] have been proposed. An equally wide range of proposals can be found regarding machine learning techniques. Commonly used classifiers that have been used for this application are: Random Forest, Neural Networks or Support Vector Machines, among others [18, 29, 38].

Those studies were carried out using speech recordings obtained using high-grade equipment like professional microphones and sound cards. Several feature datasets that have been extracted from recordings obtained with this type of equipment are publicly available [24, 30, 44]. Some authors have performed cross-database tests, which involve different microphones, environment, and even languages [35, 54], although always under controlled conditions. In this article, the term “controlled conditions” refers to the fact that there is professional supervision of the recordings and a certain control on the acoustic environment so that at least the noise level is low.

Systems built on recordings based on professional equipment are limited in the range of potential applications. Due to the ever-increasing penetration of smartphones, using these mobile devices would allow for extending the application of automatic PD detection through voice on a larger scale. The use of these devices to record phonations and build databases is an interesting strategy introduced in some recent studies. Almeida et al. [1] proposed a comparison of two different datasets of sustained vowel phonations. These datasets have been obtained through simultaneous recordings by using a professional microphone and a smartphone. Afterwards, a common methodology, consisting of preprocessing, feature extraction, and classification, was applied to both datasets comparing the results obtained in each case. In a similar way, Rusz et al. [42] simultaneously recorded different vocal tasks with a professional head-mounted condenser microphone and a smartphone, comparing the results. The outcomes point in the direction that detection of speech abnormalities due to PD via a smartphone is possible.

As the use of mobile phones increases the scope of this research line, specialized app development is a natural step. Some reviews have been published on the existing and potentially useful apps for PD patients available in the leading app stores [23, 39]. However, they concluded that, despite the clear potential of this type of technology, further efforts and more improvements are needed for it to be effectively used in a real clinical scenario. In line with this demand, a smartphone app frontend in conjunction with a computing server backend has been designed and implemented as a necessary step to build a mobile-assisted voice condition analysis system. The app allows patients to provide data and physicians to check the complete record of every patient. The system is completed with a machine learning approach to perform PD detection on the server side. This approach is built on top of a feature extraction process that includes some of the most relevant algorithms for PD detection, a recursive feature elimination selection process, and a classifier. To provide robust results cross-validations have been considered. Besides, approaches with six different classifiers have been implemented for comparison purposes. The system also allows its use with future implementations to aid also disease monitoring.

A critical aspect is to check the results obtained in increasingly realistic environments. The works previously mentioned were issued in a controlled environment and under supervision. More concretely, in Rusz et al. [42] the speech recordings were performed in a quiet room with an environmental noise level lower than 50 dB, and with a specialist who guided the participants through the recording protocol. In the case of Almeida et al. [1], the recordings were taken in a sound-proof booth. However, there are also recent studies that use public repositories where participants send their voice recordings and complementary information (age, health status, sex, etc.) without any professional supervision. This is the case of mPower PD database [7]. Some previous contributions using this database show the results of applying different feature extraction and machine learning techniques to perform PD detection based on uncontrolled conditions, that is, unknown acoustic environment and without a professional control to make sure that the recordings strictly follow the protocol [48, 49, 55, 56]. These studies do not ensure age and sex balances in the mPower-based datasets they use. Age and sex balances are necessary to avoid potential biases in the results. Also, to the authors’ best knowledge, cross-database studies that use data obtained in a realistic environment have not been presented. Research that considered smartphone recordings has focused on datasets collected either in controlled or uncontrolled conditions. However, both types of scenarios have not been jointly considered under the same methodology.

The research hypothesis is that the accuracy obtained by a mobile-assisted PD detection system based on voice tested on a controlled scenario (in terms of acoustic environment and professional supervision) is degraded when the scenario is uncontrolled. The aim of this research work is to analyze the impact of uncontrolled acoustic environment and lack of professional supervision during the recordings avoiding the influence of the feature extraction and machine learning algorithms. This requires the application of exactly the same methodology on controlled and uncontrolled databases and the realization of cross-database experiments, in which the training is performed with one database and the test with the other one.

One of the databases is an in-house one (UEX database), collected with professional supervision in a controlled environment. It has been obtained from an experiment specifically conducted to help in the detection of PD. The second one is a subset of the public mPower database, collected in a realistic environment without professional control. This subset has been chosen to ensure age and sex balance as well as comparable disease severity in relation to the in-house database. The concrete voice recordings from mPower study that we have used can be checked in the Appendix, which provides the health codes, unique identifiers provided by mPower. Both databases are also the same size. The comparison allows for evaluation of the performance degradation that might be expected when moving an automatic PD detection system from a controlled mobile scenario to an uncontrolled one. Also, cross-database tests are performed to assess the generalizability of the results.

The novel contributions of this paper can be summarized as follows:

- Performance comparison of a speech-based PD detection approach on two different databases created by using smartphones, one of them recorded under controlled conditions (quiet acoustic environment, professional supervision) and the other one collected without supervision in realistic environments (mPower-based database).
- Cross-database experiments involving the controlled database and the database recorded in realistic environments.
- Methodologically robust analysis based on the following considerations: balanced datasets regarding age and sex, comparable disease stage between datasets, identical methodology (preprocessing, feature extraction, feature selection and six classification algorithms) applied in all the experiments.
- Design and implementation of client–server system architecture: Android-based app and artificial intelligence engine, ready to perform further analysis in semi-controlled clinical trials.

## Results

### Experimental settings

The methodology proposed in Section is applied to the UEX and mPower-based databases. A total of 100 iterations of stratified 5-fold cross-validations have been used for the feature selection step. For hyperparameter optimization with grid search also a stratified 5-fold cross-validation has been issued. Finally, the classification process consists of 1000 iterations. In each one of them the set is randomly split in training and test subsets with a 75–25% ratio stratified by health status.

### Results for UEX database

Table 1 shows the evaluation metrics resulting from applying the machine learning approaches with the considered specifications to the UEX database.

**Table 1:** Evaluation metrics (mean ± standard deviation) obtained with the proposed procedure by using the UEX database

	Accuracy rate	Sensitivity	Specificity	AUC
Gradient Boosting	0.7503 ± 0.0983	0.7683 ± 0.1486	0.7331 ± 0.1697	0.8387 ± 0.0964
Logistic Regression	0.8897 ± 0.0820	0.9007 ± 0.1145	0.8788 ± 0.1324	0.9627 ± 0.0522
Passive Aggressive	0.9205 ± 0.0723	0.9396 ± 0.1005	0.9018 ± 0.1108	0.9756 ± 0.0403
Perceptron	0.9083 ± 0.0781	0.9284 ± 0.1030	0.8881 ± 0.1232	0.9713 ± 0.0457
Random Forest	0.7631 ± 0.1024	0.7666 ± 0.1591	0.7605 ± 0.1486	0.8787 ± 0.0821
SVM	0.9148 ± 0.0853	0.9229 ± 0.1102	0.9076 ± 0.1229	0.9749 ± 0.0483

Three out of six approaches (Passive Aggressive, Perceptron, and Support Vector Machine (SVM)) produced accuracy rates greater than 0.9, and Logistic Regression is close to this value. Random Forest and Gradient Boosting showed a downgrade in performance with accuracy rates around 0.75. Sensitivity and specificity are used to analyze how balanced the system is by checking whether PD or healthy subjects are better detected. All of the approaches provided slightly larger sensitivities (right classifications for subjects suffering from PD) than specificities (right classifications for healthy people). However, these differences are small and it can be concluded that all of them are reasonably well balanced.

**Fig. 1:**
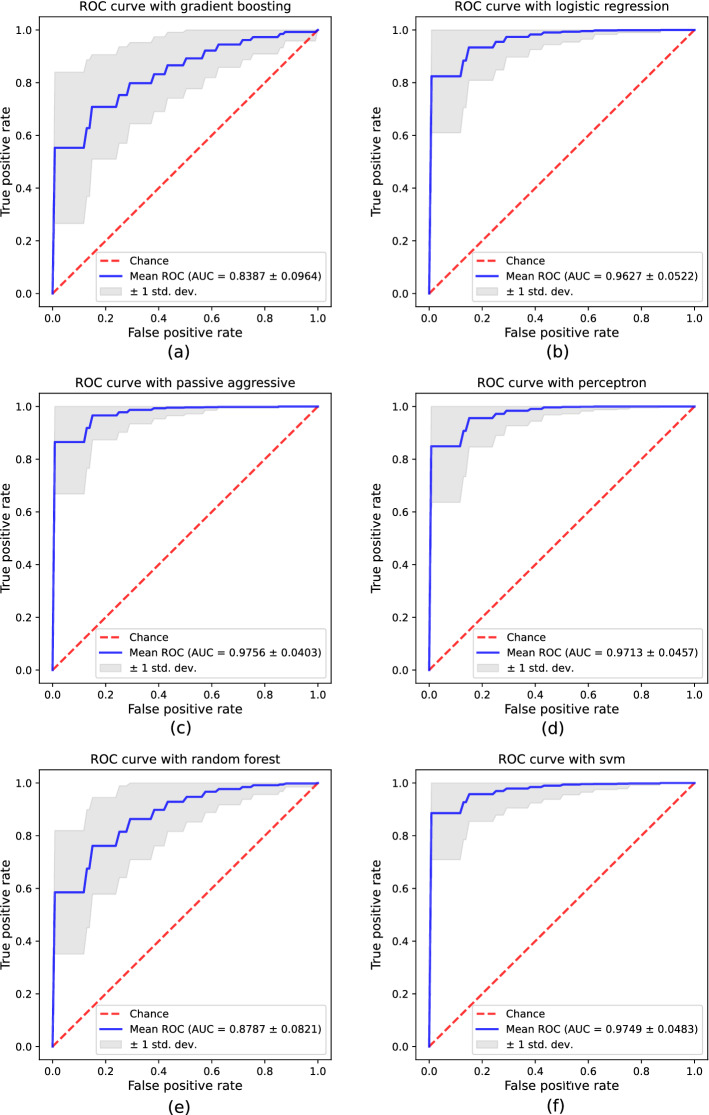
ROC curves and AUC metric obtained with the proposed procedure by using the UEX database: **a** Gradient Boosting, **b** Logistic Regression, **c** Passive Aggressive, **d** Perceptron, **e** Random forest, **f** Support Vector Machine

Figure 1 shows mean receiver operating characteristic (ROC) curves (blue lines) with bands for ± 1 standard deviation (light gray area) for the six classifiers under consideration. The ROC curve shows the trade-off between false-positive rate (FPR = 1-specificity) in the *x*-axis and true-positive rate (TPR=sensitivity) in the *y*-axis. As performance is measured with the area under the curve (AUC) metric, ROC curves closer to the top-left corner indicate a better performance.

Gradient Boosting ROC curve presented in Fig. 1a provides a relatively good AUC mean value of 0.8387 with a standard deviation of 0.0964. Given the shape of the curve the results are far from the optimal classifier (TPR = 1, FPR = 0), and the slow growth indicates that we should face a very high FPR for TPR higher than 0.7. Random Forest ROC in Fig. 1e shows a similar performance, with mean AUC = 0.8787, and the same problem of high FPR for TPR higher than 0.7. On the other side, Logistic Regression (Fig. 1b), Passive Aggressive (Fig. 1c), Perceptron (Fig. 1d) and SVM (Fig. 1f) show a great AUC, well above 0.95 in every case, and a standard deviation that shows a perfect classifier for some of the cross-validation experiments performed. In these cases, the FPR/TPR trade-offs are much more beneficial, with FPR lower than 0.2 for TPR above 0.9 in every case.

Table 2 presents the run times separated by feature selection, grid search and classification. The most time-consuming task for all six classifiers is feature selection, since a very exhaustive recursive feature elimination with cross-validation (RFECV) has been applied, followed by grid search. Finally, classification, applied here with cross-validation, is the least expensive task in terms of computational time. Gradient Boosting and Random Forest, which yield the lowest performance, also have the largest execution times. The rest of the classifiers have closer values, all of them with less than one minute for the total run time.

**Table 2:** Run times in seconds for the different steps of the proposed procedure by using the UEX database

	Feature selection	Grid search	Classification	Total
Gradient Boosting	390.05	318.47	105.69	814.21
Logistic Regression	24.63	21.51	12.28	58.42
Passive Aggressive	23.43	11.97	12.21	47.61
Perceptron	22.17	7.58	12.37	42.13
Random Forest	938.81	286.77	155.31	1380.89
SVM	17.75	14.00	9.16	40.91

Table 3 summarizes the results from the feature selection process, providing a global perspective about which features are the most relevant for each approach. Checking the number of times each feature has been selected, it can be determined that Lempel–Ziv complexity (LZ-2), Cepstral Peak Prominence (CPP), Period Density Entropy (RPDE), and 4th and 8th Mel Frequency Cepstral Coefficients (MFCC) are the most selected features. Specifically, the most chosen feature is LZ-2, which is the only one selected by all the approaches. Conventional features like Jitter, Shimmer or HNR are not very relevant. Gradient Boosting only selected three features, but it performs badly in accuracy metrics and run time results. The rest of the classifiers selected a similar number of features and chose the five most relevant ones (LZ-2, CPP, RPDE, MFCC4, and MFCC8).

**Table 3:**
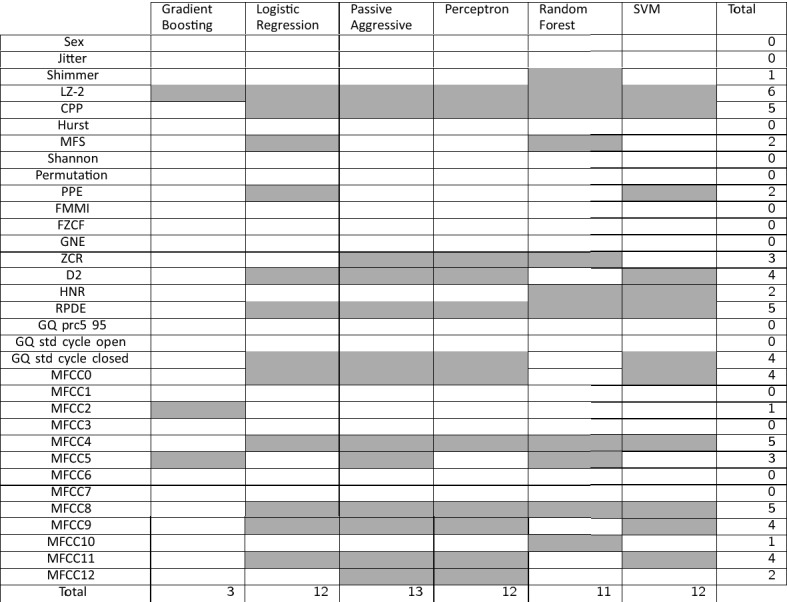
Selected features for each classifier in the proposed procedure by using the UEX database

In summary, the best result is obtained with the Passive Aggressive approach. It produces the largest accuracy rate (0.9205) and AUC (0.9756), with the lowest standard deviations (0.0723 and 0.0403, respectively). Besides, its computing time is low. SVM and Perceptron approaches are also very competitive in accuracy metrics and computing time. Any of these three approaches could be considered for the mobile-assisted system to detect PD.

### Results for mPower-based database

The same experimental settings and methodology applied to UEX database is applied to this matched database based on mPower study. Table 4 presents the accuracy metrics. T-tests reported statistically significant differences (${p\text{-values}}<0.001$) for comparisons of each accuracy metric and method between UEX database and mPower-based database.

**Table 4:** Evaluation metrics (mean ± standard deviation) obtained with the proposed procedure by using the mPower-based database

	Accuracy	Sensitivity	Specificity	AUC
Gradient Boosting	0.7138 ± 0.1051	0.7419 ± 0.1712	0.6868 ± 0.1665	0.7560 ± 0.1147
Logistic Regression	0.6523 ± 0.1101	0.6530 ± 0.1961	0.6525 ± 0.1910	0.7330 ± 0.1258
Passive Aggressive	0.6167 ± 0.1167	0.6096 ± 0.2168	0.6247 ± 0.2141	0.6935 ± 0.1349
Perceptron	0.6245 ± 0.1179	0.6334 ± 0.2211	0.6164 ± 0.2150	0.6923 ± 0.1411
Random Forest	0.6957 ± 0.1048	0.7123 ± 0.1659	0.6823 ± 0.1664	0.7475 ± 0.1110
SVM	0.6562 ± 0.1122	0.6476 ± 0.2047	0.6657 ± 0.1879	0.7437 ± 0.1240

Accuracy rates are much lower than in the case of UEX database, ranging from 0.6167 to 0.7138. The best approach is based on Gradient Boosting classifier. This means that the accuracy rates have been degraded for all the approaches. In percentage terms, the reductions with respect to UEX database range from 4.9% to 33.0%. Analogously, sensitivities and specificities are also degraded, with reductions ranging from 3.4% to 35.1%, and from 6.3% to 30.7%, respectively. Sensitivities and specificities are close for most of the approaches when applied to mPower dataset.

**Fig. 2:**
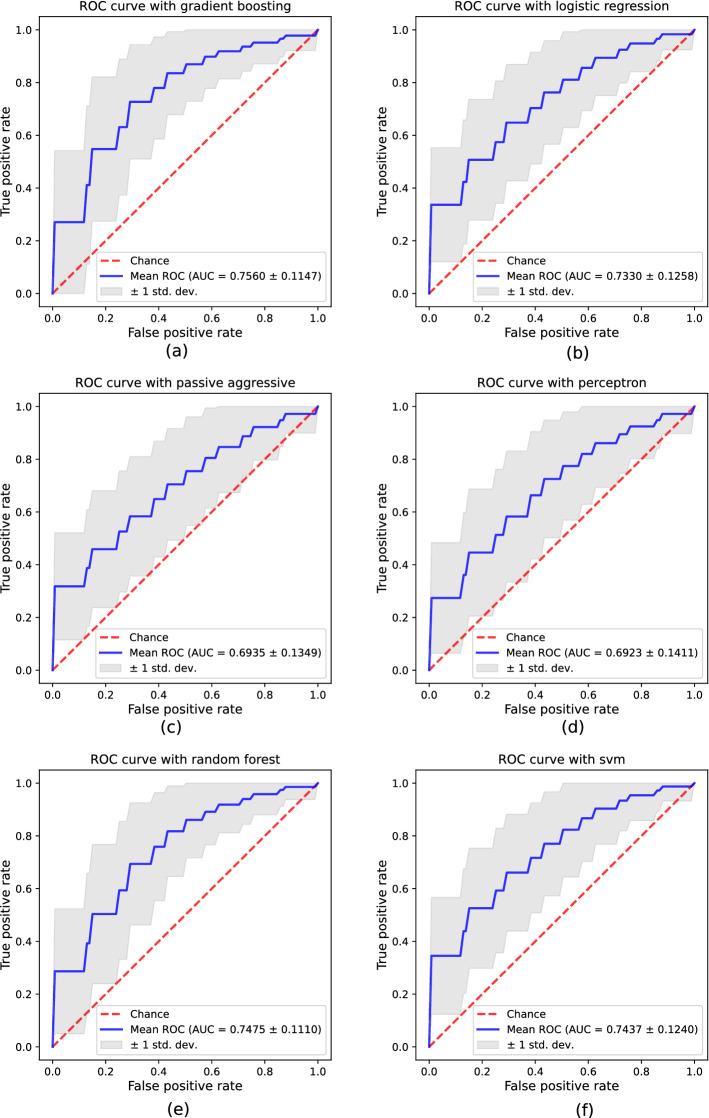
ROC curves and AUC metric obtained with the proposed procedure by using the mPower-based database: **a** Gradient Boosting, **b** Logistic Regression, **c** Passive Aggressive, **d** Perceptron, **e** Random forest, **f** Support Vector Machine

Figure 2 shows the ROC curves (blue lines) with bands for ± standard deviation (light gray area). Superiority of the ROC curves in Fig. 1 with respect to Fig. 2 can be seen at a glance. Following the AUC criterion, the best approach is also Gradient Boosting, but its AUC value is only 0.7449. In fact, the AUC values range from 0.6923 to 0.7560, which means reductions of AUC between 9.9% and 28.9% with respect to the UEX database. Every classifier but Gradient Boosting (Fig. 2a) produces an AUC under 0.75, though the latter slightly exceeds that value, making it the best option. In every case, the trade-off between FPR and TPR is quite low. It is worth noting that the curve does not reach TPR = 1 in any case, no matter the threshold. Also, Passive Aggressive and Perceptron (Fig. 2c and d) are near random classification, given that standard deviation shows that, in the worst cases, AUC stays as low as 0.5.

It is remarkable that the standard deviations of the metrics are greater in the case of mPower-based database in spite of the fact that the mean values are lower than those of the UEX database. This means that the approaches provide more dispersed values with mPower-based dataset, and therefore the results obtained with UEX dataset are more robust.

With respect to the computing time, the results match those obtained with UEX database. Table 5 shows the computing times separated by tasks. There are two approaches, Gradient Boosting and Random Forest, that have large computing times. The other four approaches keep their execution time below one minute for the whole process.

**Table 5:** Run times in seconds for the different steps of the proposed procedure by using the mPower-based database

	Feature selection	Grid search	Classification	Total
Gradient Boosting	392.43	379.88	24.64	796.95
Logistic Regression	19.22	15.81	9.58	44.60
Passive Aggressive	18.27	8.82	8.99	36.09
Perceptron	17.08	5.48	9.62	32.18
Random Forest	939.55	281.49	82.51	1303.55
SVM	18.61	13.59	9.21	41.42

Finally, it is remarkable that the feature selection processes have provided different results than those of UEX database. Table 6 shows the selected features for each approach. Sex, Shimmer, MultiFractal Spectrum Width (MFSW), Glottal Quotients (GQ prc5-95 and GQ std cycle open), and MFCC6 have been the most selected features, being Shimmer selected by all the approaches. MFCCs have also been selected with UEX database. The number of selected features range from 7 to 10.

**Table 6:**
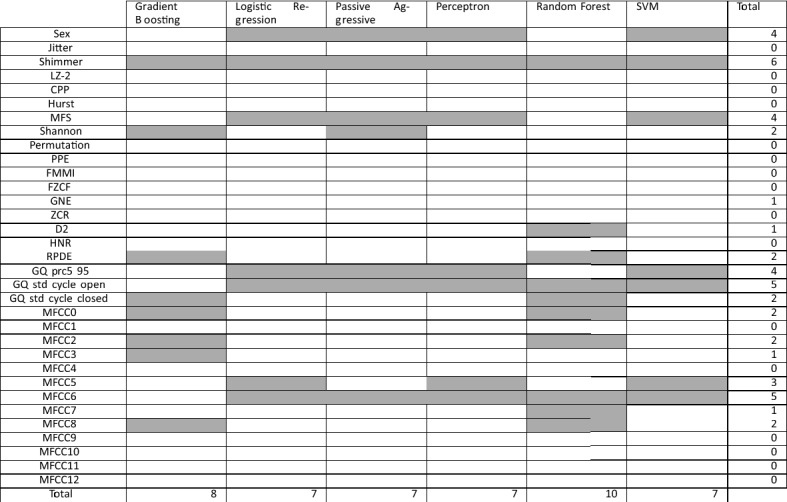
Selected features for each classifier in the proposed procedure by using the mPower-based database

### Cross-database tests

In this type of experiments, we use the selected features and hyperparameter values obtained in a single-database experiment and test the performance on the other database.

Table 7 shows the results obtained when the selected features and hyperparameter values obtained from UEX database are applied on mPower-based database. It can be observed that the detection capability has been lost, with a result close to random classification. Specifically, the degradation can be quantified with a reduction percentage with respect to the results obtained with the UEX database in 27.7–45.5% for accuracy, 31.0–49.9% for sensitivity, 30.0–43.1% for specificity, and in 33.7–48.4% for AUC. This indicates that it is not recommendable to train the system with a controlled database if it is going to be applied on an uncontrolled scenario.

**Table 7:** Evaluation metrics (mean ± standard deviation) obtained by selecting features and hyperparameter values from UEX database and testing the performance on mPower-based database

	Accuracy	Sensitivity	Specificity	AUC
Gradient Boosting	0.5234 ± 0.1139	0.5358 ± 0.1827	0.5131 ± 0.1912	0.5377 ± 0.1294
Logistic Regression	0.5380 ± 0.1233	0.5376 ± 0.2024	0.5393 ± 0.2036	0.5569 ± 0.1495
Passive Aggressive	0.5021 ± 0.1243	0.4706 ± 0.2092	0.5357 ± 0.2130	0.5036 ± 0.1548
Perceptron	0.5289 ± 0.1205	0.5267 ± 0.2019	0.5334 ± 0.2027	0.5522 ± 0.1452
Random Forest	0.5519 ± 0.1245	0.5286 ± 0.1956	0.5818 ± 0.1980	0.5822 ± 0.1474
SVM	0.5230 ± 0.1209	0.5308 ± 0.2023	0.5166 ± 0.2025	0.5442 ± 0.1432

The results obtained using the reverse procedure are shown in Table 8. In this case, the selected features and hyperparameter values are obtained from the mPower-based database and tested on UEX database. Now, the reduction percentage with respect to the results obtained with the mPower-based database are in 7.7–13.6% for accuracy, 5.7–11.6% for sensitivity, 6.3–24.2% for specificity, and 8.3–21.5% for AUC. In spite of the low performance, the results are better than in the previous experiment. This indicates that system robustness is increased when a variety of acoustic conditions is used to determine the feature set and hyperparameter values, and they are applied to voice recordings fulfilling a very strict recording protocol.

**Table 8:** Evaluation metrics (mean ± standard deviation) obtained by selecting features and hyperparameter values from mPower-based database and testing the performance on UEX database

	Accuracy	Sensitivity	Specificity	AUC
Gradient Boosting	0.6165 ± 0.1046	0.6260 ± 0.1786	0.6089 ± 0.1736	0.6664 ± 0.1216
Logistic Regression	0.6022 ± 0.1175	0.5940 ± 0.2138	0.6114 ± 0.1985	0.6495 ± 0.1426
Passive Aggressive	0.5302 ± 0.1262	0.5877 ± 0.2529	0.4738 ± 0.2426	0.5446 ± 0.1625
Perceptron	0.5877 ± 0.1258	0.5925 ± 0.2219	0.5849 ± 0.2142	0.6322 ± 0.1539
Random Forest	0.6421 ± 0.1003	0.6717 ± 0.1749	0.6152 ± 0.1664	0.6851 ± 0.1216
SVM	0.6053 ± 0.1142	0.6033 ± 0.2062	0.6074 ± 0.2024	0.6511 ± 0.1416

## Discussion

In this study, we have proposed a methodology to discriminate PD patients from healthy subjects based on sustained phonations of /a/ vowel recorded by a smartphone. We applied feature extraction, data standardization, feature selection, hyperparameter optimization, and six different classification techniques. The results obtained when applying this methodology to recordings obtained under controlled conditions (protocol supervised by specialized staff, same recording room and same smartphone) have been presented first.

Under these controlled conditions, the procedure has allowed to identify a set of features that provide good performance using accuracy, sensitivity, specificity and AUC metrics. The results demonstrate the relevance of LZ-2 and RPDE. The high ability for PD discrimination of these and other features based on nonlinear dynamics has been noted by other authors (see e.g., Orozco-Arroyave et al. [36]). It is also remarkable the role played by CPP which, as opposed to classic features such as Jitter, can be robustly extracted even from strongly aperiodic signals like those obtained from PD patients with a severely affected voice. It is also known the huge potential of MFCCs for different classification applications based on speech. They have been previously used for PD detection by Sakar et al. [45]. MFCCs allow for capturing differences in the resonant characteristics of the vocal tract. It has been reported that patients with PD present an asymmetric centralization of tongue position during the phonation of vowels, which produces a decrease in the vowel space area in comparison to healthy speakers [2]. This can explain the high number of MFCCs present in the subsets of selected features that result from our study.

With UEX database, the best results have been achieved using Passive Aggressive classifier: 0.9205 in accuracy rate, 0.9396 in sensitivity, 0.9018 in specificity, and 0.9756 in AUC. Placing these results in the context of the literature is a complex task since a real comparison of methodologies would require working on the same databases, or at least on databases with comparable disease stages which also ensure age and sex balance. To the authors’ best knowledge the published scientific work does not allow for a comparison that fulfills these three requirements. However, in the next paragraphs we provide a rough overview of the performance obtained using professional microphones and smartphones.

In the case of professional microphones, in a recent work, Solana-Lavalle et al. [46] compare their accuracy rate (0.94) with other scientific works presenting values between 0.85 and 1. In the case of databases based on smartphone recordings, Almeida et al. [1] use sustained vowel recordings and a similar methodology than ours: feature extraction and classification process with 2/3 training and 1/3 test ratio for cross-validation. They achieve 0.9294 of accuracy rate and 0.9240 of AUC by using 1-nearest neighbor classifier with smartphone recordings. The health status of PD patients was evaluated at stages 1 to 2.5 according to HY (Hoehn and Yahr) scale. The experimental design was not age-balanced, since the mean age of PD patients was 61.5 years, while the mean age of healthy subjects was 41.8 years. Rusz et al. [42] recorded different vocal tasks including sustained vowels with a professional microphone and a smartphone. The experiment was well balanced in terms of age and sex. The mean HY stage was 2.1 (0.4) in comparison to 2.7 (0.53) in this study. Their methodology is based on the extraction of 6 acoustic features and the use of Logistic Regression with Leave-One-Out cross-validation for classification. They achieved an AUC of 0.85 for smartphones. Zhang [57] proposed a smartphone-based PD detection service by using a deep learning methodology based on stacked autoencoders and K-Nearest-Neighbor classifier achieving a maximum accuracy value of 0.9881. However, this can not be considered a complete smartphone-based system since their experimental results were not obtained from recordings made by mobile phones. Instead, they used already extracted features from publicly available datasets.

Once the potential of our methodology to perform automatic detection of PD has been proved on a controlled scenario, the next step is applying the same techniques in an uncontrolled one, therefore, we considered mPower database [7]. It must be pointed out that this database has been massively collected. As a consequence, it contains some faulty recordings that would not pass a simple playback quality check performed by the majority of the users if they were immersed in a real clinical scenario. Also, it includes some inconsistencies in diagnosis, having recordings from the same subject labeled as PD affected and healthy. In order to issue a valid comparison with it, a previous work has been done to select recordings from the database which provided a balanced set by sex, age, and disease stage. The results show a best accuracy rate of 0.7138 with sensitivity of 0.7419, specificity of 0.6868 and AUC of 0.7560 for Gradient Boosting versus a best accuracy rate of 0.9205 with sensitivity of 0.9396, specificity of 0.9018 and AUC of 0.9756 for Passive Aggressive with the UEX database. This has provided statistically significant differences for the four accuracy metrics (${p\text{-values}}<0.001$). This shows a clear degradation in the accuracy performance in comparison to UEX database that is not only reported for the best methods, but for all ones. In this case, using mPower-based database produces a performance degradation of 22.5% for accuracy rate, 21% for sensitivity, 23.8% for specificity and 22.5% for AUC.

The aforementioned difficulties arise again when these results are intended to be placed in the context of the scientific literature, because previous works based on mPower database do not use exactly the same subset of recordings. Since the database has been massively collected, experiments based on large cohorts have been performed. For example, with a subset of mPower database consisting of 2222 phonation recordings, 933 PD patients and 1289 healthy subjects, Giuliano et al. [14] obtained AUC values over 0.82 in the discrimination of PD subjects from healthy ones. Their methodology was based on Neural Networks and Logistic Regression models. Wroge et al. [55] reached a maximum accuracy rate of 0.86 by using Minimum Redundancy Maximum Relevance for feature selection and Gradient Boosted Decision Tree for classification, with a total of 5826 voice recordings. Tougui et al. [48] achieved an accuracy rate of 0.9578 by using Least Absolute Shrinkage and Selection Operator feature selector, hyperparameter tuning, and Extreme Gradient Boosting classifier with 18210 recordings (9105 PD patients and 9105 healthy subjects). In these works based on large cohorts, sex and age balances between PD and healthy groups are not ensured in the experiments.

The application of an identical methodology to both databases has allowed for checking the differences that can be expected when moving from a controlled scenario to an uncontrolled one. As previously mentioned, a clear degradation in the detection performance can be noted, but there are also differences concerning the selected features and the best classifier. In terms of selected features, with the exception of Gradient Boosting, the results obtained with UEX database show a good stability when varying the classification method. A similar conclusion regarding stability across classifiers can be extracted from the results obtained on mPower-based database, which means that the database plays a more important role than the classification method. On mPower-based database the most relevant features are: Sex, Shimmer, MFSW, GQ std cycle open, GQ prc5 95 and MFCC6. Although the features are different for each database, we can identify some common aspects. For example, if we consider the most repeated features, in both cases the role is shared by features that are able to capture source-related irregularities considering the classical source-filter theory of speech production (CPP in the case of UEX database, GQ std cycle open and GQ prc5 95 in the case of mPower-based database), resonance-related features (MFCCs) and features based on nonlinear analysis (LZ2 and RPDE in the case of UEx database and MFSW in the case of mPower-based database).

A limitation of our work is the size of the databases. The reason is the difficulty in recruiting people suffering from PD in the case of the controlled database (UEX database). Nevertheless, 60 people (30 with PD and 30 healthy controls) is a reasonable size compared to other studies in the scientific literature. For example, in Benba et al. [4] the number of participants is 40 (20 with PD and 20 healthy); in Little et al. [24], this number was 31 (23 with PD) and in Novotny’ et al. [34] the total number was 80 (40 with PD and 40 healthy).

Regarding computation time, the executions on both databases yield similar conclusions in the comparison of classifiers. In a real clinical application, the first two tasks will be only applied from time to time to improve the learning process, so that both the selected features and the searched hyperparameters will be used during a long time. Furthermore, the third task, classification, is applied here with cross-validation, but in real time approaches it will be applied only to the new subject. For all these reasons, computation time is not a critical issue. Anyway, even for model assessment purposes, the experiments have been performed in a very reduced time.

Due to the differences in the selected features found in the single-database experiments, we have performed cross-database tests, in which the feature set obtained for each classifier with one database has been applied to the other one. Although we observe an important degradation in performance in both cases, the results are slightly better when feature selection is performed on mPower-based database and applied to UEX database than when using the reverse procedure. The wide variety of acoustic conditions available in mPower database due to the fact that the recordings were performed by the participants themselves is considered a strength that could be exploited to achieve robustness. However, it must be taken into account that, since this database has been massively collected, some information provided by the participants may be incorrect and some voice recordings may be of bad quality, having an impact on the performance. Some research initiatives point out that personalized medicine and collaboration between patients and health professionals might provide a greater insight in disease impact by allowing patients to provide and self assess their condition outside clinical environment [22]. Therefore, a semi-controlled scenario appears as a very suitable option. This means that the participants would submit their audio files, recorded by following a strict recording protocol in a variety of acoustic conditions, but the clinical information is provided by the physicians. The proposed mobile-assisted system is considered a very useful tool to address this semi-controlled scenario.

## Conclusion

Smartphones have a great potential to assist diagnosis and improve patient monitoring of many diseases. In the case of PD, smartphones allow for an easy collection of speech waveforms that can be used with clinical purposes. This can help general practitioners to conduct triage and neurologists or movement disorders specialists to perform diagnosis and tracking. In particular, PD management could be highly benefited by smartphone-based systems, due to different aspects such as increasing incidence, diagnosis prone to errors, difficulty of tracking progression, and the fact that it mostly targets elderly people, which in general have more difficulties to visit a hospital, among others.

We have designed and implemented a mobile-assisted voice condition analysis system based on an Android app frontend in conjunction with a machine learning-based implementation hosted on a computing server backend. Although the machine learning approach is focused on a detection task, the app allows for monitoring PD progression.

The most relevant novel contribution of our work is that we have applied identical methodology to an in-house smartphone-based database recorded under controlled conditions (in a quiet room with low noise level and with professional supervision of the recordings) and to a subset of mPower database (created by collecting data from free-living scenarios). This comparison of results is performed within a methodologically robust framework ensuring age and sex balance and comparable disease stage. The results of this study show the potential of the proposed system under controlled conditions. The performance decreases when testing the methodology with the uncontrolled database and strongly drops in cross-database tests.

These results confirm the research hypothesis and suggest that semi-controlled scenarios have high potential to be useful in real clinical applications. In these semi-controlled scenarios the relevant clinical information is provided by the physicians. Also, general practitioners (in the context of triage for diagnosis) or patient and caregivers (in a PD monitoring application...) should receive some initial training after which a test should be mandatory to ensure that the speech protocol is fully understood and that the user has some control on the acoustic environment regarding noise level. Within this framework, recordings would be submitted via smartphone from different environments.

Future analyses should be performed on new datasets obtained in the described semi-controlled clinical scenarios. The proposed app is a very suitable tool for this task because it allows patients to submit phonations and physicians to check the complete record of every patient. In those semi-controlled conditions, also longitudinal studies would be interesting for PD tracking. This type of studies are difficult to perform because they require larger amounts of time. However, they would be very useful to achieve optimal treatment of PD.

## Methods

A mobile-assisted voice condition analysis system for PD detection is proposed. This system is built through the design and implementation of a mobile application that communicates with a server backend to collect and process voices recorded following a protocol. The system extracts acoustic features from the voice recordings and use them to feed machine learning approaches specifically designed for a PD detection task. An experiment has been conducted to test the proposed approaches. Also, the same architecture was used on a different database collected using smartphones and results are compared. In this section, the several parts that compose the system are described.

### System architecture and mobile app design

**Fig. 3:**
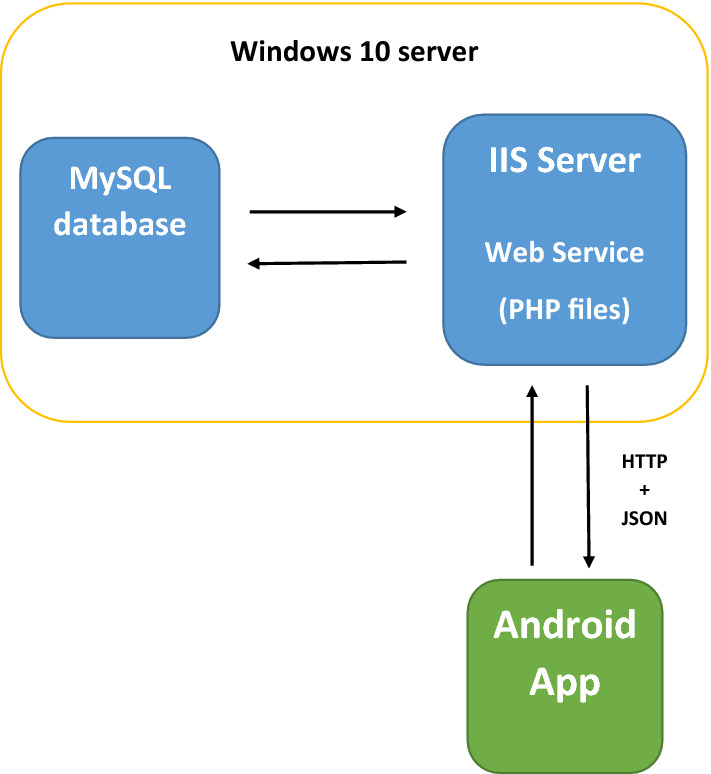
System structure

Voice recordings are received and stored in a server where they can be accessed and processed. The server runs Windows 10 and the Windows Internet Information Services (IIS) functionality has been employed to host a web service written in PHP that manages a MySQL database. An Android app exchanges information with the server via an HTTP connection, using JavaScript Object Notation (JSON) format to organize it. Figure 3 shows the system structure in a schematic way.

The Android application has two types of user accounts: patients and doctors. Every user needs to fill a registration form with the most relevant personal information, some of which will be used for the authentication. This form is slightly different for patient and doctor accounts. The user receives a notification in the email account provided after the registration process. It is necessary to give permission for the use of personal data as part of a non-profit study. In the patient case, as part of the registration process, an informed consent document is requested to be signed by accepting participation in the mentioned study. Users can sign the document through the phone’s touch screen.

**Fig. 4:**
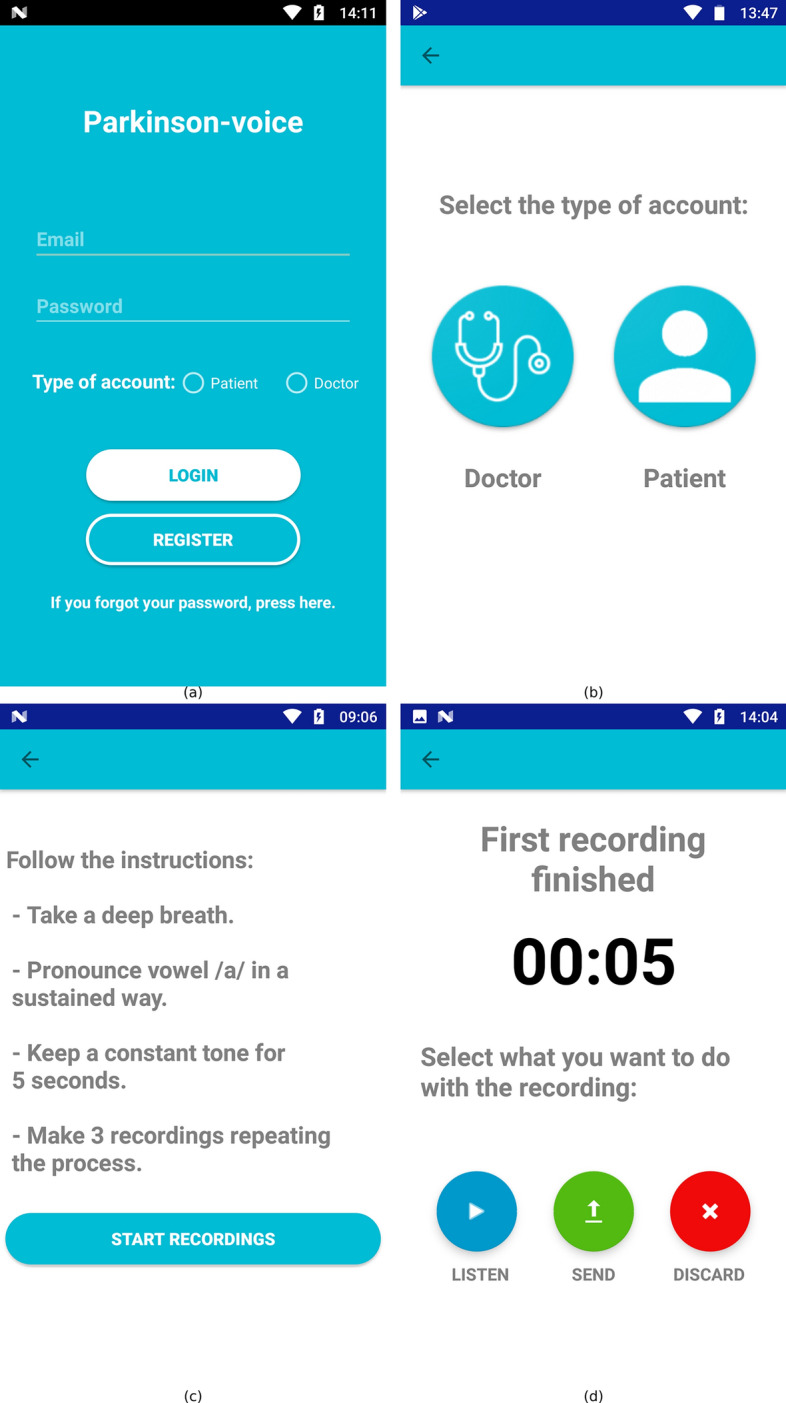
Screenshots obtained from the Android mobile application: **a** first screen, **b** types of accounts, **c** instructions, **d** recording process

Once completely registered, users can employ their credentials (email and password) to access the functionalities allowed for the type of account created. On the one hand, patient accounts are able to record and send audio files following the given instructions. After each recording, the user can choose between three options: submit to the server, listen or discard and try again. On the other hand, doctor accounts can associate patients with their account to keep track of their cases. Only doctor accounts can access patient data, and only those linked to the doctor account. Figure 4 shows some screenshots extracted from the app: Fig. 4a shows the registration and login screen of the system; Fig. 4b shows the screen that allows to select the type of account (patient or physician) in the registration process; Fig. 4c presents the screen showing the instructions the patient should follow to perform the recordings; after that, three possibilities (listen, send, discard) are offered to the patient, as shown in Fig. 4d.

### Participants

Two databases were used in the study. The first one was generated by the University of Extremadura with the collaboration of the Regional Association for Parkinson’s Disease of Extremadura (UEX database). A total of 60 participants with ages between 51 and 87 years old were recruited, 30 of whom were affected by PD (PD subjects) and 30 were healthy. Patients suffering from PD were recruited among the voluntary members of the Regional Association for Parkinson’s Disease of Extremadura that meet the following inclusion criteria: (1) have a definitive diagnosis of PD; (2) medical reports available. After the voluntary PD patients were recruited, then the healthy group was selected to approximately match sex and age. Healthy subjects were selected with the requirement of neither having been diagnosed with PD nor having any symptom related to PD. Those not meeting the inclusion criteria were not eligible for participation. There were 24 men and 6 women in the PD group and 26 men and 4 women in the healthy group. The mean (standard deviation) of the age was 70.27 (9.54) for the PD group and 67.33 (8.57) for the healthy group. The mean time in years since diagnosis was 9.93 (6.16), and the mean time in hours since the last medication dose was 2.21 (1.32). The mean HY stage was 2.6 (0.4). The research protocol was approved by the Bioethics Committee of the University of Extremadura. All of them signed an informed consent.

The second database (mPower-based database) is a subset extracted from the mPower Public Researcher Portal, a mobile PD study [7]. The goal of this initiative is to collect information of patients suffering from PD. The objective is to describe more precisely the experience, habits, lifestyle, drawbacks, and interactions with medication of those patients. By using a mobile application, each volunteer records different aspects of the impairment caused by the disease and tracks their evolution. The study is open to anyone who wants to participate, and the only requirement is having a personal iPhone for PD patients, and also not having been diagnosed for the control group subjects. These requirements are not checked.

The subjects selected to build the mPower-based database were matched with the ones from the UEX database by keeping exactly the same proportion of health status and sex, and approximately the same age, so the results can be compared. Specifically, the mean of the age was 68.36 (8.14) for the PD group and 65.23 (7.76) for the healthy group. The mean time in years since diagnosis was 7.83 (4.54), whereas the estimated mean HY stage was 2.7 (0.53). The mean time since the last medication dose was not available. The voice recordings were stored for posterior use. Table 9 shows the codes of these voice recordings extracted from mPower.

### Recording task and equipment

The selected vocal task was sustained phonation of /a/ vowel due to several advantages, such as its wide spread use in the scientific literature; simplicity to realize by the participants, which avoids fatiguing them, especially in the case of patients with more advanced PD stages; ease of analysis and control; ubiquity in different languages; and the fact that it is unaffected by phonetic context or intonation [12].

The recording task for UEX database consists of performing three 5-seconds voice phonations, pronouncing the /a/ vowel in a continuous and uninterrupted way holding pitch and loudness as constant as possible.

Due to the biological variability, voice recordings from a particular subject result in similar but not identical waveforms. The consequence is that the features are also not identical when extracted from different recordings from the same individual. To obtain more stable predictors, it was decided to record three utterances per subject so that the feature values can be later averaged to produce an only feature vector per subject.

All the voice recordings were made using the same smartphone (model BQ Aquaris V) at a sample frequency of 44.1 kHz. The recordings were taken at the facilities of the Regional Association for Parkinson’s Disease of Extremadura (Spain), always in the same room, that was relatively quiet but did not have any special acoustical isolation. A specialized person was present to ensure that all the participants properly followed the voice recording protocol and registered the complementary information based on medical reports.

Voice recordings from mPower were performed on participants’ iPhones (4th generation or a more advanced version) or iPods (5th generation or newer) by using the /a/ vowel phonation protocol. A sample frequency of 44.1 kHz was used. Since participants record themselves without supervision, this database includes a variety of acoustic environments. They were also responsible to fill in the form including the complementary information, which makes the obtained data somehow unreliable.

Before applying feature extraction, all the recordings from both databases were trimmed down to one second discarding any leading or trailing silence. This length has been considered sufficient to extract speech features from sustained vowel phonations by other authors [40]. Voice recordings were edited using Audacity software (release 2.0.5).

### Feature extraction

The same feature extraction algorithms are applied to both databases. A total of 33 features have been considered to measure different aspects related to speech production: Sex (male, female), Jitter, Shimmer [51], CPP [34], HNR, glottal-to-noise excitation ratio, zero crossing rate [3], 3 GQ features [45], MFCCs (13 features) [52], correlation dimension, RPDE, pitch period entropy [51], Hurst’s exponent, LZ-2 [36], permutation entropy, Shannon’s entropy, first minimum in mutual information [25], MFSW [17], first zero in correlation function [16]. The methods have been coded in Python.

Considering these feature extraction algorithms, 180 vectors (60 subjects $\times$ 3 audio recordings/subject) of 34 feature components (health status plus extracted features) were initially stored in a spreadsheet for UEX database. This spreadsheet was reduced to 60 vectors of 34 features by aggregating every 3 vectors corresponding to the same subject through a component-wise average. This ensures that each subject is represented by only one feature vector and no artificial increase of the dataset is considered. In the case of the mPower-based database, 60 vectors of 34 feature components were stored in another spreadsheet. These datasets were used to feed the machine learning approaches.

### Statistical methods

Due to the amount of features, many of them measured in different scales, a preprocessing step is required. A standardization was applied based on the mean and standard deviation of each feature.

Several classifier methods have been considered to test their performance in this context. They cover a wide range of techniques commonly used in machine learning applications such as linear methods (Logistic Regression [33]), ensemble decision trees (Random Forest [9]), neural networks (Perceptron [31]), online learning (Passive Aggressive [32]), additive models (Gradient Boosting [21]) and separating data models (SVM [13]).

In order to compare the performance of the procedure with each classifier, and based on the confusion matrix, the following metrics have been considered: accuracy, sensitivity, specificity, and AUC. Student’s t-test for independent samples were applied to report statistically significant differences between mean values of accuracy metrics. P-values smaller than 0.05 were considered statistically significant.

**Fig. 5:**
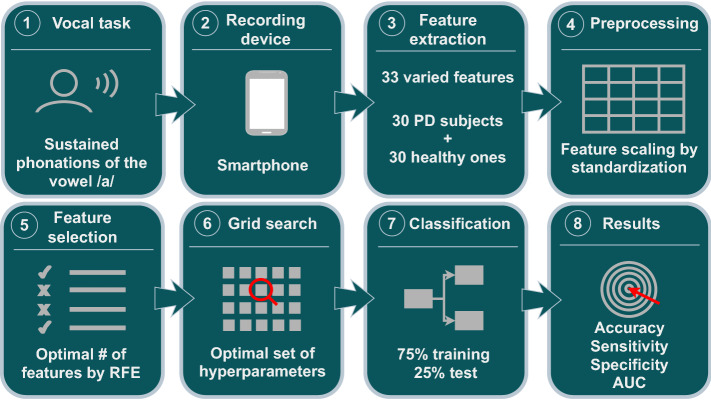
Methodology followed in the procedure

Figure 5 represents the whole procedure. After preprocessing, the machine learning approaches contain 3 steps: Feature selection, hyperparameter optimization, and classification process (steps 5, 6 and 7). The involved techniques have been coded in Python based on the scikit-learn package [15]. Next paragraphs provide a detailed description of these three steps.

*Feature selection* Once having a standardized dataset, a feature selection process is applied. RFECV [27] is used to eliminate redundant features while keeping a good classification performance. The algorithm trains the chosen classifier and removes the feature with the weakest effect on the classification process, providing a feature top-ranked list based on the order of removal. It provides the optimal number of features by selecting the top-ranked features of the mentioned ranking. The process is repeated several times in order to achieve a representative value. Since the number of optimal features can vary in each iteration, the result of each iteration is stored in a vector and the value of the first quartile after all iterations is chosen as the final number of selected features. A stratified k-fold cross-validation [5] is used in the RFECV algorithm, which consists in splitting the complete dataset in *k* groups but maintaining the same ratio between PD subjects and healthy ones in each group.

*Hyperparameter optimization* Each classifier has its own parameters that can be adjusted, these are called hyperparameters. Once the most relevant features are known for the chosen classifier, a hyperparameter tuning has been issued in order to know which is the best configuration for the classifier. The method selected for this step is Grid Search [47]. It optimizes the chosen hyperparameters using stratified k-fold cross-validation again. Accuracy is calculated for each combination of classifier parameter values, selecting the set that provides the best result. These values are used in the classification process.

*Classification process* With the selected features and the optimal hyperparameter configuration for each classifier, a stratified cross-validation is issued. The dataset is randomly split into only a training and a test subset, maintaining the ratio between the number of PD and healthy subjects in each set. In order to maintain training and test sets independent from each other, the scaling is applied after this splitting with respect to the training set values. With this splitted data, the classifier is fitted with the training data and after that, it makes a prediction of the PD-healthy state given the test subset. Finally, its predictions are compared with the correct labels. Based on this comparison, the considered metrics are extracted for each iteration. In order to obtain global results, this classification process is repeated several times and the resulting accuracy metrics are averaged after all iterations are finished.

At the end, for each approach, the following are available: the selected features, the optimal hyperparameter configuration, the averaged accuracy metrics, and run times.

## Data Availability

The dataset from the in-house voice database (UEX database) is available from the corresponding author on reasonable request. The availability of data contributed by users of the Parkinson mPower mobile application are part of the mPower study developed by Sage Bionetworks and can be accessed through Synapse at https://www.synapse.org/mPower doi: [10.7303/syn4993293.

## Appendix: Codes of voice recordings from mPower

In this appendix, the codes of the voice recordings that have been considered from mPower database are presented in Table 9.

**Table 9:** Codes of considered voice recordings from mPower

Recording ID	
Healthy	PD
0f81a5ef-14d4-4a19-9d89-deabeb728adb	45155beb-a91f-4bca-8296-7612c6915af8
7c5a339d-35ba-48ec-8447-f51aec949a1e	955aa8c3-9116-43e7-9e4b-d1843be4839a
ebfb61fc-c218-4d3a-a680-eb3b4ce3b91d	4412716d-e1b0-4572-b976-8bcb7669925e
b3c61a60-acff-426b-aaeb-d8b6d4c31cb6	0ce23959-8092-47ce-b394-0f65c951a548
740240f3-6752-456b-9f39-6ede3afb3423	a86b7dee-759d-452c-86b5-4b6a248d7286
be0ecb7f-95a2-468a-a12e-2fb738c9b922	9e03615f-1f52-4a95-94bf-cc5805d0c3b8
18cd4553-1c4f-4f6d-a622-8951eb79e780	e2766ec9-e97d-4224-81a8-35b095ea9fd6
3accca87-eaf1-4219-b0e0-af29eb426093	22ad855e-1c57-4f9b-bf67-2a44f2a3ce41
f908e76b-b4e1-40b6-86a5-b4a0def0e6c0	7eac5187-e241-4f80-b704-0f91b8041dc6
75ad7180-afb1-49ea-b766-221106d32e02	0d1c8246-8e42-45e5-b662-91e26e6cb6d4
a3907344-70e3-410c-a6ac-3ae5e790d3ad	02ed9d30-620f-4c6c-88ce-64a286df79b9
393a367c-9727-4390-96f8-6a7a3c6e2797	90899edf-a289-4557-aff9-a168fd82a92e
6348a018-d039-4c38-8920-66ceba01c8e0	06e8ee83-0e3a-4575-a7e4-0c1c813376b6
2fabaecf-423b-4db1-98e6-54daf6844a2d	2b72e6d8-9963-4edd-a8ca-ae2d4262f640
8fa63734-04cb-4f15-a954-34db4d0c9d2e	eb764994-17ef-4421-b052-9acbb0440a3b
15791b9e-89c9-421b-be3c-c3acf89bd167	a9b6687a-c533-410e-8f87-c319a969b98e
4366e9a8-292c-48a2-afa2-d6cbbbf438a9	b662bb1d-ab78-479e-86c8-7fc1bd1df59d
b3277c31-add4-40ae-8621-54da00f50012	1864ea1c-b861-49c3-85f8-549ba6c04679
a467eb63-7f6b-4dee-800f-ee053f0f5d90	2e4b8613-3bab-4cb7-a569-47b52a45a3f9
dcc7e425-7b58-4a04-998a-34822c68cb81	e8a9288b-bdc1-4f09-9f7c-3937d56a4d7f
2df7b01c-d48f-404a-9c09-acdce4cab75c	303e5481-66af-4ba8-bb7e-3dfef44b588b
7c1728ca-408f-4c6c-9d28-94dd61313c65	af9163dc-93e1-4b57-9195-86f6b8ff6725
59ee208f-181e-4d67-9b1f-888cd5036e87	f20af903-16e2-413d-8826-26fc7b51ef38
a17e3358-441b-484a-bac7-868a82784cf6	c79e662a-493c-4d56-9216-b7edd9b4e682
b35db6a8-cff0-4755-9969-3a34a3fc46c7	992b993e-7de6-473a-ae08-d5048a8fb143
54d0e506-71bd-4d27-bc5a-9a360e5b1048	13ded0a1-ea81-4a5c-8895-bc442f79c3f6
5e764adf-411c-42d2-ad2c-a2ddee58abfa	52b32a74-7a52-450c-b8ad-b06020549a98
5fa385c3-e977-45df-8a26-1a41e1086c24	ded9a617-1b5f-4f55-b36c-b89aaa20c08e
8011c74a-aa69-46b3-af41-f09705dd3010	f9bf9e84-39a2-45b4-b9af-5d6e6256b4ad
4a9c103f-6e69-4b1a-a82d-7fc30dd0c488	31d0f0f6-511a-44ac-b69f-fd4b6f278502

## Abbreviations

AUC: Area under the receiver operating characteristic curve
CPP: Cepstral peak prominence
FPR: False-positive rate
FZCF: First zero in correlation function
GQ: Glottal quotient
HNR: Harmonic-to-noise ratio
HY: Hoehn and Yahr
IIS: Windows Internet Information Services
JSON: JavaScript Object Notation
LZ-2: Lempel–Ziv complexity
MFCC: Mel frequency cepstral coefficients
MFSW: MultiFractal spectrum width
PD: Parkinson’s disease
RFECV: Recursive feature elimination with cross-validation
RPDE: Recurrence period density entropy
ROC: Receiver operating characteristic curve
SVM: Support vector machine
TPR: True-positive rate
UEX: Universidad de Extremadura
